# Effectiveness of implementation strategies for clinical guidelines to community pharmacy: a systematic review

**DOI:** 10.1186/s13012-015-0337-7

**Published:** 2015-10-29

**Authors:** Kim Watkins, Helen Wood, Carl R. Schneider, Rhonda Clifford

**Affiliations:** 1 School of Medicine and Pharmacology, University of Western Australia, Perth, Australia; 2 Faculty of Pharmacy, The University of Sydney, Sydney, Australia

**Keywords:** Community pharmacy, Pharmacy, Pharmacists, Implementation, Information dissemination, Guideline, Evidence-based medicine

## Abstract

**Background:**

The clinical role of community pharmacists is expanding, as is the use of clinical guidelines in this setting. However, it is unclear which strategies are successful in implementing clinical guidelines and what outcomes can be achieved. The aim of this systematic review is to synthesise the literature on the implementation of clinical guidelines to community pharmacy. The objectives are to describe the implementation strategies used, describe the resulting outcomes and to assess the effectiveness of the strategies.

**Methods:**

A systematic search was performed in six electronic databases (Medline, EMBASE, CINAHL, Web of Science, Informit, Cochrane Library) for relevant articles. Studies were included if they reported on clinical guidelines implementation strategies in the community pharmacy setting. Two researchers completed the full-search strategy, data abstraction and quality assessments, independently. A third researcher acted as a moderator. Quality assessments were completed with three validated tools. A narrative synthesis was performed to analyse results.

**Results:**

A total of 1937 articles were retrieved and the titles and abstracts were screened. Full-text screening was completed for 36 articles resulting in 19 articles (reporting on 22 studies) included for review. Implementation strategies were categorised according to a modified version of the EPOC taxonomy. Educational interventions were the most commonly utilised strategy (*n* = 20), and computerised decision support systems demonstrated the greatest effect (*n* = 4). Most studies were multifaceted and used more than one implementation strategy (*n* = 18). Overall outcomes were moderately positive (*n* = 17) but focused on process (*n* = 22) rather than patient (*n* = 3) or economic outcomes (*n* = 3). Most studies (*n* = 20) were rated as being of low methodological quality and having low or very low quality of evidence for outcomes.

**Conclusions:**

Studies in this review did not generally have a well thought-out rationale for the choice of implementation strategy. Most utilised educational strategies, but the greatest effect on outcomes was demonstrated using computerised clinical decision support systems. Poor methodology, in the majority of the research, provided insufficient evidence to be conclusive about the best implementation strategies or the benefit of clinical guidelines in this setting. However, the generally positive outcomes across studies and strategies indicate that implementing clinical guidelines to community pharmacy might be beneficial. Improved methodological rigour in future research is required to strengthen the evidence for this hypothesis.

**Protocol registration:**

PROSPERO 2012:CRD42012003019.

**Electronic supplementary material:**

The online version of this article (doi:10.1186/s13012-015-0337-7) contains supplementary material, which is available to authorized users.

## Background

In the last 30 years, there has been a major shift in healthcare towards evidence-based medicine and the use of clinical guidelines to facilitate evidence-based practice [1–4]. Clinical guidelines are defined as “systematically developed statements to assist practitioner and patient decisions about appropriate healthcare for specific clinical circumstances” [5]. Their primary aim is to improve patient care and, ultimately, patient health outcomes [4]. They also have many potential benefits for health professionals including improved clinical decision-making and consistency of care [4].

**Table 1:** Summary of included studies evaluating implementation of clinical guidelines in community pharmacy

First author, year	Country	Sample size	Time frame	Clinical area (guidelines)	Methodology for collecting outcome data	Key findings for main outcome (++, +, minimal effect, variable results, no evidence of effect)
Curtain, C. et al, 2011 [54]	Australia	Community pharmacies, Total (*n* = 185), Intervention (*n* = 73), Control (*n* = 112)	12 weeks	Proton pump inhibitors (National Prescribing Service proton pump inhibitor dosage recommendation)	Clinical intervention software data, Prescription data, Patient survey, Economic data	++
De Almeida Neto, A.C. et al, 2000 [55]	Australia	Pharmacists, Total (*n* = 43) from community pharmacies, Total (*n* = 30), Intervention, (*n* = 15 pharmacies), Control (*n* = 15 pharmacies)	Three-week baseline data collection followed by a 6-week intervention period, immediately after the workshop	Non-prescription drugs—analgesics	Simulated patient methodology, Pharmacist survey	+
				(Protocol for non-prescription medicines with a focus on identifying inappropriate “off-label” use of compound analgesics)		
De Almeida Neto, A.C. et al, 2001 Study 1 [56]	Australia	Community pharmacies, Total (*n* = 24), Intervention group (*n* = 16), Control group (*n* = 8)	Three 4-week periods: before and immediately	Non-prescription drugs—analgesics	Simulated patient methodology	++
			after a 3-h training workshop, and after a further interval of 14 weeks	(Protocol for non-prescription medicines with a focus on identifying inappropriate “off-label” use of compound analgesics)		
De Almeida Neto, A.C. et al, 2001 Study 3 [64]	Australia	Not stated	Three 2-week periods (baseline, post workshop1 and post workshop2)	Non-prescription drugs—cough and cold medicines	Simulated patient methodology	++
				(Protocol for non-prescription cough and cold medicines)		
De Almeida Neto, A.C. et al, 2001 Study 5 [68]	Australia	Pharmacists and pharmacy assistants from community pharmacies, total (*n* = 99)	12 weeks of pseudo-patron and feedback visits, post a training visit	Non-prescription drugs—heartburn and indigestion treatments (protocol for heartburn management)	Simulated patient methodology	++
De Almeida Neto, A.C. et al, 2001 Study 5A [69]	Australia	Not stated	Not mentioned in paper	Non-prescription drugs—analgesics		
				(Protocols specific for analgesics)		
Egen, V. et al, 2003 [70]	Germany	Gynaecologists, total (*n* = 311), interviewed baseline (*n* = 24), post-intervention (*n* = 27), pharmacists, total (*n* = 418), baseline (*n* = 21), post (*n* = 21), women in childbed: baseline (*n* = 131), post (*n* = 118)	16 months intervention with interviews pre and post	folic acid (The Societies of Nutrition, Gynaecology and Obstetrics, Human Genetics, Paediatrics, and Neuropaediatrics jointly issued corresponding recommendations)	Simulated patient methodology, Patient interview, Gynaecologist telephone interview	No evidence of effect
Guirguis, L.M. et al, 2007 [71]	Canada	Practicing pharmacists, Total (*n* = 8) tested the diabetes tool	Participants were introduced to the tools, and their experience was evaluated after 2 weeks. One year later a survey was faxed to investigate any sustained use/change in practice	Diabetes	Pharmacist self-report forms. Pharmacist survey, Focus group discussion	+
				(Canadian Diabetic Guidelines)		
Koster, E.S, et al, 2014 [72]	The Netherlands	Community pharmacies, Total (*n* = 78), Pharmacists, (*n* = 95) and technicians (*n* = 337) were interviewed, dispensing data—only available for (*n* = 52) pharmacies	Dispensing data was collected for the period between 1 Jan. 2008 to 10 May. 2011.	Methotrexate (Safe Methotrexate Dispensing Recommendations published by the Royal Dutch Pharmaceutical Society in accordance with the Dutch Health Care Inspectorate)	Pharmacist-structured interviews, Electronic dispensing records	+
Kradjan, W.A. et al, 1999 [57]	USA	Community pharmacies, Total (*n* = 90)	Intervention period 4th	Asthma	Patient survey	No evidence of effect
		Intervention, (*n* = 44)	March 1996 to 30th June 1996	(Current asthma treatment guidelines)		
		Control, (*n* = 46)				
Legrand, S.A. et al, 2012 [67]	Belgium	Pharmacists, Total (*n* = 100)	Intervention pharmacies completed a baseline questionnaire, and after a 6-month intervention period participants (including controls) were asked to complete a post-questionnaire	Medicines and driving	Pharmacist survey	+
		(IS) intervention group (*n* = 68), (SA) intervention group (*n* = 12), Control group (*n* = 20)		(DRUID (driving under the influence of drugs, alcohol and medicines project) dispensing guidelines)		
Martin, B.A. et al, 2010 [73]	USA	Pharmacists, Total (*n* = 25)	The study was conducted during 2002–2003	Smoking cessation	Pharmacist survey	+
				(National tobacco cessation guidelines (Treating Tobacco Use and Dependence: Clinical Practice Guideline), which incorporates the 5A’s counselling process)	Pharmacist telephone interview	
					Invoices submitted—(remuneration claims)	
Naunton, M. et al, 2004 [65]	Australia	GPs, total (*n* = 200)—74 % visited	Baseline data collection	Osteoporosis	Pharmacist survey	+
		Community pharmacies, total (*n* = 69)—100 % visited pharmacists, total (*n* = 81) to complete surveys	(Mar–Sept 2001) Intervention mail out (Oct 2001) Detailing visits (Jan–May 2002), post intervention data collection (Mar–Sept 2002)	(Locally produced guidelines adapted from American College of Rheumatology, UK Consensus Group and Osteoporosis Australia guidelines on the management of glucocorticoid induced osteoporosis)	GP survey	
					Hospital admission data	
					Prescription data—(remuneration claims)	
Patwardhan, P.D. et al, 2012 [58]	USA	Intervention group:	The research was carried out from July 2008 until March 2009 with a 1-month study period in November 2008	Smoking cessation	Pharmacist self-report forms	+
		Community pharmacies (*n* = 8), Pharmacists (*n* = 16), Technicians (*n* = 24)		(Treating tobacco use and dependence: Clinical practice guideline (2008 update)	Quit-line referral reports (from an external agency)	
		Control group		The specific recommendation to use AAR in situations in which the 5A’s approach may not be feasible)		
		Community pharmacies (*n* = 8),				
		Pharmacists (*n* = 16), Technicians (*n* = 24),				
Puumalainen, I. et al, 2005 [74]	Finland	Pharmacists, Total (*n* = 734)	TIPPA implementation, 4 years (2000–2003).	Guideline-based counselling	Pharmacist survey	Minimal effect
			Data collection for this research, 1 month—June 2002	(The United States Pharmacopeia (USP) Medication Counselling Behaviour Guidelines disseminated through a 4-year project (TIPPA))		
Raisch, D.W. 1998 [59]	USA (New Mexico)	Community pharmacies, Total (*n* = 301)	Ketorolac claims records were reviewed for 3 months before intervention (Aug–Oct 1995) and for 3 months after intervention (Dec–Feb 1996)	Ketorolac	Dispensing data	+
		Intervention (*n* = 150), Control (*n* = 151)		(Manufacturers prescribing guidelines for ketorolac)	Economic data	
		Data obtained from:				
		Community pharmacies (*n* = 167), Intervention (*n* = 90)				
		Control (*n* = 77)				
Reeve, J.F. et al 2008 [60]	Australia	Community pharmacies, Total (*n* = 52), Pharmacists, Total (*n* = 150) recruited to attend training	6-week study period where the computer-generated prompt was active plus another 2-week period where interventions were recorded but the prompt was deactivated	Diabetes	Clinical intervention software data, Prescription data, Pharmacist survey	++
		Intervention (*n* = 31) pharmacies		(American Diabetes Association- Clinical practice recommendations. Aspirin therapy in diabetes. Recommendation for the addition of low-dose aspirin therapy to medication regimen of high-risk patients with diabetes)		
		Control (*n* = 21) pharmacies				
Sigrist, T. et al, 2002 [66]	Switzerland	Community pharmacies, Total (*n* = 27)	2 months	Non-prescription drugs	Simulated patient methodology	Variable results
		Intervention (*n* = 14)		(Personalised advice protocol based on change and health belief models and used in assessment of appropriate use of non-prescription medications)		
		Control (*n* = 13),				
		intervention participants to attend workshops,				
		Pharmacists (*n* = 20),				
		Pharmacy assistants (*n* = 65),				
Thorley, T. et al, 2006 [75]	UK	Community pharmacies, Total (*n* = 1222)	March 2003 (initial implementation communication), mystery shopping data collected over 4 months (May–Aug 2003)	Asthma	Simulated patient methodology	+
				(Evidence-based questions (×3) from Royal College of Physicians (RCP) to determine patient asthma control and to direct response based on answers)		
Van de Steeg-van Gompel, C. et al 2011 [61]	The Netherlands	Community pharmacies, Total (*n* = 71) grouped into 36 clusters	Sept 2006–Feb 2008	Statins drugs	Prescription data	No evidence of effect
		Intervention (*n* = 37) (18 clusters), Control (*n* = 34) (18 clusters)		(Protocol for Education at First Dispensing of a Statin (EAFD) and Protocol for Education at Second Dispensing of a Statin (EASD))	Pharmacist self-report forms	
					Pharmacist telephone interview,	
Watson, M.C. et al, 2002 [62]	UK	Community pharmacies, Total (*n* = 60)	Mar–Apr 2000 baseline data July–Nov 2000 post intervention data collection	Non-prescription	Simulated patient methodology, Pharmacist survey, Economic data	No evidence of effect
		EO intervention (*n* = 15), CPE intervention (*n* = 15), EO and CPE intervention (*n* = 15), Control (*n* = 15)		Drugs—vulvovaginal candidiasis		
				(Evidence-based guidelines for OTC treatment of vulvovaginal candidiasis)		
Watson, M.C. et al, 2007 [63]	UK	Community pharmacies, Total (*n* = 20)	The intervention comprised two training sessions 1 month apart (Sept and Oct 2005)	Good pharmacy practice	Simulated patient methodology	No evidence of effect
		Medication care assistants (*n* = 30)		(Royal Pharmaceutical Society of Great Britain (RPSGB) guidelines and WWHAM guideline. Professional and good practice guidelines for the supply of non-prescription medicines)	Pharmacist survey	
		Intervention (*n* = 20 MCAs), Control (*n* = 10 MCAs)				

Key:

Simulated patient methodology: this involves data collection using covert patients (mystery shoppers) to assess pharmacy practice [95]

**Table 2:** Comparison of studies

		Curtain, C. 2011	De Almeida Neto, A.C. 2000	De Almeida Neto, A.C. 2001 Study 1	De Almeida Neto, A.C. 2001 Study 3	De Almeida Neto, A.C. 2001 Study 5	De Almeida Neto, A.C. 2001 Study 5A	Egen, V. 2003	Guirguis, L.M. 2007	Koster, E.S. 2014	Kradjan, W.A. 1999	Legrand, S.A. 2012	Martin, B.A. 2010	Naunton, M. 2004	Patwardhan, P.D. 2012	Puumalainen, I 2005	Raisch, D.W. 1998	Reeve, J.F. 2008	Sigrist, T. 2002	Thornley, T. 2006	Van de Steeg-van Gompel, C.H. 2011	Watson, M.C. 2002	Watson, M.C. 2007
Study design	Randomised controlled trial (RCT)	✓	✓	✓							✓				✓		✓	✓			✓	✓	✓
	Non-randomised controlled trial				✓								✓						✓				
	Controlled before and after studies											✓											
	Other					✓	✓	✓	✓	✓			✓			✓				✓			
	Participants self-selected	✓	✓	✓	✓	✓	✓		✓	✓	✓	✓	✓		✓	✓		✓	✓		✓	✓	✓
Participants included in the intervention	Pharmacists	✓	✓	✓		✓	✓	✓	✓	✓	✓	✓	✓	✓	✓	✓	✓	✓	✓	✓	✓	✓	
	Pharmacy support staff (technicians and assistants)			✓	✓	✓	✓			✓					✓				✓	✓	✓	✓	✓
	Other health professionals							✓							✓								
	Patients/Public							✓								✓							
Intervention type (*EPOC taxonomy)*	Educational materials	✓						✓		✓	✓	✓	✓	✓	✓	✓	✓			✓	✓	✓	
	Educational meetings		✓	✓	✓	✓	✓				✓		✓		✓	✓			✓		✓		✓
	Educational outreach visits							✓						✓								✓	
	Mass media Campaign							✓															
	Audit and feedback		✓	✓	✓	✓	✓												✓				
	Reminders	✓									✓	✓						✓					
	Practice support		✓	✓					✓						✓			✓			✓	✓	
	Fee for service										✓												
Basis for intervention	Improve clinical knowledge				✓	✓	✓	✓		✓	✓	✓	✓	✓			✓			✓	✓	✓	
	Improve communication skills		✓	✓	✓				✓						✓	✓			✓	✓	✓		✓
	Based on addressing organisational culture								✓						✓								
	Based on a specified theory of behaviour change		✓	✓									✓		✓				✓				✓
	Based on addressing identified barriers (tailored)																				✓		✓
Outcome measures	Practitioner outcomes—subjective measures		✓						✓	✓	✓	✓	✓	✓	✓	✓		✓			✓	✓	✓
	Practitioner outcomes—objective measures	✓	✓	✓	✓	✓	✓	✓		✓			✓	✓	✓		✓	✓	✓	✓	✓	✓	✓
	Secondary measures (practitioner or patient)		✓						✓		✓	✓	✓	✓		✓		✓			✓		
	Patient health outcomes	✓					✓			✓													
	Economic outcomes	✓															✓					✓	
Effectiveness	Overall effective in achieving main outcomes	✓	✓	✓	✓	✓	✓		✓	✓		✓	✓	✓	✓		✓	✓		✓			

Key:

Educational materials: distribution of educational materials to support guideline-based practice (paper-based, electronic, patient focused, practice tools), disseminated by mail, email or in person. Educational meetings: conferences, lectures or workshops

Educational outreach visits: use of a trained person to meet with health professionals to give information with the intent of changing the professional’s practice, includes academic detailing

Mass media: varied use of communication that reaches a large number of people including television, radio, newspapers etc. targeted at a population level. Audit and feedback: any summary of clinical performance, which may also include recommendations for clinical action

Reminders: interventions involving computer prompts to support practice

Practice support: follow-up contact (e.g. visits or phone calls) to provide motivation and support to practitioners post education

**Table 3:**
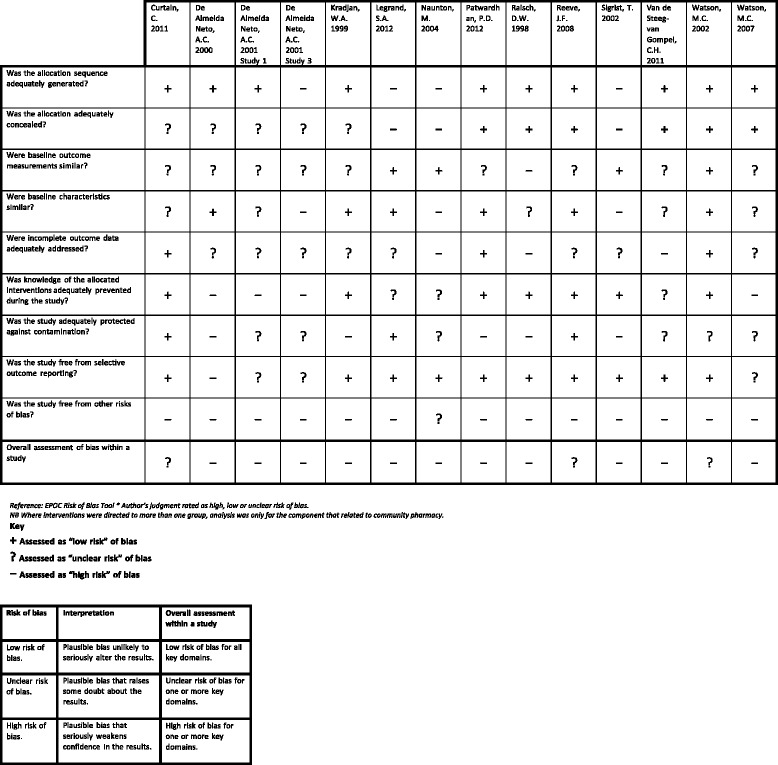
Results of risk of bias assessment using the EPOC risk of bias tool for RCTs, NRCTs, CBA studies

**Table 4:** Results of risk of bias assessment using the Newcastle-Ottawa risk of bias tool for cohort studies

		De Almeida Neto, A.C. 2001 Study 5	De Almeida Neto, A.C. 2001 Study 5A	Egen, V. 2003	Guirguis, L.M. 2007	Koster, E.S. 2014	Martin, B.A. 2010	Puumalainen, I. 2005	Thornley, T. 2006
Selection	Representativeness of the exposed cohort	–	–	*	–	–	–	*	*
	Selection of the non-exposed cohort	*	*	*	*	*	*	*	*
	Ascertainment of the exposure	*	*	*	–	*	–	–	*
	Demonstration that outcome of interest was not present at start of the study	*	*	*	–	*	*	–	–
Comparability	Comparability of cohorts on the basis of the design or analysis								
Outcome	Assessment of outcome	*	*	*	–	*	–	–	*
	Was follow-up long enough for outcomes to occur	–	–	*	*	*	*	–	–
	Adequacy of follow-up cohorts	–	–	*	–	–	–	–	–

Reference: Newcastle-Ottowa Quality Assessment Scale

NB, where interventions were directed to more than one group; analysis was only for the component that related to community pharmacy

Key

Based on a star system (*) with a range of 0 to 9 stars possible. Three domains are tested:

1. Selection of study groups (up to one star allowed for each item)

2. Comparability of the groups (up to two stars allowed)

3. Outcomes (up to one star allowed for each item)

**Table 5:**
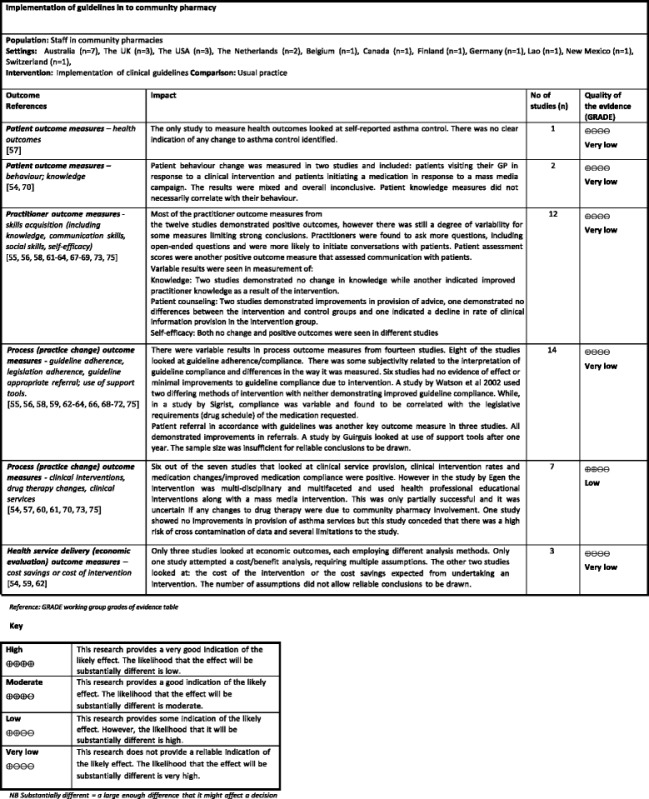
Summary of outcomes and quality of evidence using GRADE

**Fig. 1:**
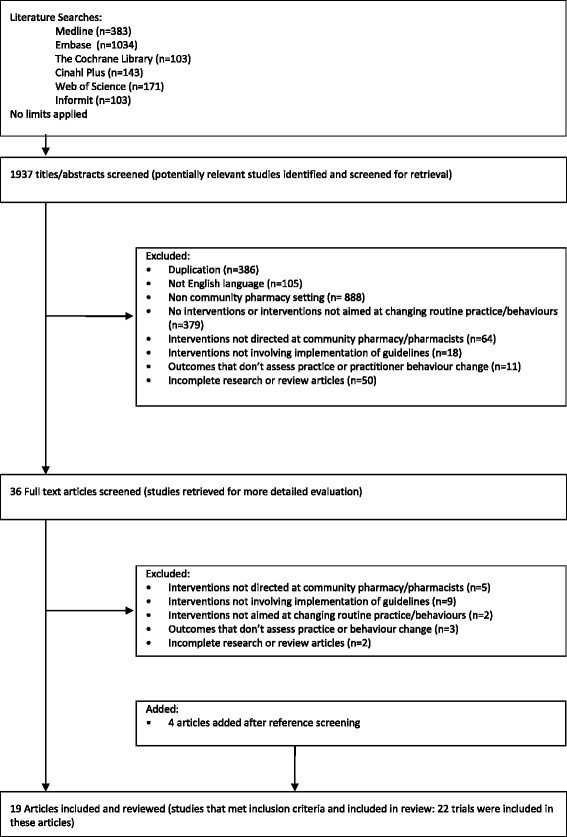
Flow diagram of study selection

While research has demonstrated that clinical guidelines can achieve improvements in health professional practice and patient outcomes, there is substantial variability in observed effectiveness [2, 6]. A recent study in Australia reported that patients receive appropriate, evidence-based care, on average, only 57 % of the time [7]. Despite the proliferation of clinical guidelines, there are still barriers in translating the evidence in clinical guidelines into practice across all healthcare settings [8, 9]. This has resulted in an increase in research attempting to identify the factors that influence successful clinical guideline implementation [10].

Development and dissemination of clinical guidelines does not necessarily translate to health professional uptake and adherence [1, 11]. The impact of clinical guidelines depends on the characteristics of the guidelines themselves as well as the implementation strategies employed [12]. Characteristics such as the complexity of guidelines and the evidence base used in their development are important factors influencing uptake into clinical practice [1]. However, the evidence regarding effective implementation strategies is less clear, and in some instances contradictory, as seen in the conflicting conclusions of research in this field [1, 2, 8, 11, 13]. For example, it is still unclear whether multifaceted implementation strategies, that target multiple barriers to implementation or use multiple strategies, are more effective than single strategies, despite the commonly held assumption that a multifaceted approach is superior [1, 2, 14, 15].

These contradictions have led to the recommendation that implementation strategies should be based around a clear rationale, such as overcoming barriers to change (tailored implementation interventions) [14, 16]. However, methodology on how to determine barriers and how to design strategies to address them, is less clear [17, 18]. Recently, the literature regarding guideline implementation has also advocated the use of theoretical frameworks to inform implementation strategies [19–22]. While theory has not been routinely used in implementation research to date, this approach is gaining momentum [23].

Most of the research into guideline implementation has been conducted with medical practitioners and in the hospital setting [1, 2, 24–26]. In the systematic review of guideline dissemination and implementation by Grimshaw, medical practitioners alone were the target of 74 % of interventions [2]. There have been a small number of reviews looking at guideline implementation for allied-health practitioners, but only a few studies relate to pharmacists, and many were in the hospital setting [13, 16, 22].

Understanding the impact of clinical guidelines in community pharmacy is important, given the expanding role of community pharmacists in primary healthcare. It is acknowledged that community pharmacists are often a patient’s first point of contact with the health system, and in some instances, the only health professional to see a patient [27]. Worldwide, community pharmacy practice is gradually evolving to incorporate the provision of clinical services and a greater focus on patient care [28]. In response to these practice changes, there has been an increase in the development of clinical guidelines for use in this setting. However, little is known about the implementation of clinical guidelines in community pharmacy.

The aim of this systematic review is to synthesise the literature on implementation of clinical guidelines to community pharmacy. The objectives are to:

- Describe the types of implementation strategies utilised.
- Describe the outcomes resulting from guideline implementation at a:
  - Practitioner level: process outcomes measuring community pharmacy practice
  - Patient level: patient outcomes measuring clinical and/or humanistic outcomes
  - Health system level: economic outcomes
- Assess the effectiveness of the implementation strategies.

## Methods

The methodology used in conducting this systematic review was in accordance with the Preferred Reporting Items for Systematic Reviews and Meta-Analyses (PRISMA) statement and checklist, using the accompanying explanation and elaboration document [29, 30]. The review protocol was registered with the International Prospective Register of Systematic Reviews (PROSPERO) on 27 September 2012 and updated in May 2015 (registration number CRD42012003019) [31].

### Search strategy and study selection

After consulting with a librarian from the Medical and Dental Library at the University of Western Australia, a comprehensive search strategy was devised. Search terms and medical subject headings (MeSH headings) chosen were those relevant to “community pharmacy” AND “clinical guidelines” AND “implementation” *(*Additional file 1*—Search terms).* The search was conducted in six electronic databases (Medline, EMBASE, CINAHL, Web of Science, Informit and Cochrane Library) up to and including the 9 November 2014. No restrictions were placed on the search.

The search results from each of the databases were collated. Articles retrieved that were not printed in English were removed along with duplicate results. The decision to remove articles not written in English was based on resource limitations. There is evidence to suggest this was unlikely to introduce systematic bias into this review[32]. Articles were then screened for eligibility based on the previously devised PICO (participants, interventions, comparators, outcomes) framework [33]. Initially titles and abstracts were screened. Full-text screening was undertaken in articles meeting the inclusion criteria or where the abstract provided insufficient information. A final list of articles was identified for inclusion in the review. Two authors (KW and HW) independently conducted the full-search strategy and eligibility assessment. Discrepancies in study selection were resolved through discussion, and where consensus could not be achieved, by mediation with a third author (CS).

### Eligibility criteria

Studies were not excluded based on research design. Experimental, quasi-experimental, intervention and observational studies were all eligible for inclusion. Studies were selected based on the PICO (participants, interventions, comparators, outcomes) framework [33] outlined below.

### Participants

Studies were included if the intervention was in the community pharmacy setting. This included interventions directed to professional staff (pharmacists, interns (unregistered pharmacists) and pharmacy students) and pharmacy support staff (pharmacy assistants, pharmacy technicians). The definition of a community pharmacy used was “a retail business registered for the provision of pharmaceutical services and from which goods and services relating to the provision of pharmaceutical services may be available to the public” [34]. Not included in the review were studies set in hospital pharmacies, multidisciplinary medical clinics run by health funds or other private organisations, outpatient clinics, consultant pharmacist services and Accredited Drug Dispensing Outlets (ADDOs).

### Interventions

Of interest were interventions involving the implementation of clinical guidelines. Studies were included if they reported dissemination and implementation strategies directed to community pharmacy which aimed to influence behaviour of pharmacists, and/or other staff, towards uptake and adherence of guideline-based practice. Studies were excluded if they were “patient programmes” and not aimed at influencing practice behaviours in the community pharmacy setting*.* This included interventions where pharmacists were instructed to provide services for the course of the project, but the aim was not to make this “usual practice” for the pharmacist. For example, the paper by Armour and colleagues in 2007, that described a guideline-based asthma management programme [35].

The description of the intervention strategies was a modified version of the Cochrane Effective Practice and Organisation Care Review Group (EPOC) taxonomy’s section on implementation strategies [36]. This included educational materials, educational meetings, educational outreach visits, mass media campaign, audit and feedback, reminders, practice support and fee for service [36]. Also investigated was the basis for the interventions. Interventions were considered “tailored” if barriers to implementation were initially identified and then addressed in the development of the implementation strategy [36, 37]. “Based on theory” was the term for interventions that used a documented theory, model or framework for the design of the intervention [20]. Interventions addressing “organisational culture” represent those interventions that focus on changing some aspect of the community pharmacy as an organisation [36, 38]. These include many constructs such as leadership effectiveness, workflows within the organisation, staffing and time management issues.

The operational definition of clinical guidelines was “systematically developed statements to assist practitioner and patient decisions about appropriate healthcare for specific clinical circumstances” [5, 36]. This definition was chosen on the basis that it has been included in the updated EPOC taxonomy [36] and also been used in two similar reviews with a similar scope, but in different practice settings [1, 16]. In the application of this definition, it was acknowledged that some studies would be excluded, for example, studies that looked at the implementation of practice standards that did not include guidance on clinical activities [39, 40] and articles that looked at policy documents and frameworks [41, 42]. One of the key determinations from the definition was that the guidelines needed to relate to “specific clinical circumstances.” The use of two reviewers and a moderator provided methodological rigour in application of the definition.

### Comparators

For most of the studies in this review, the primary comparator was “usual practice”. Usual practice is indicated by no implementation strategy being directed to the community pharmacy to change current practice.

### Outcomes

Outcomes were based on the EPOC classification scheme and included patient outcomes, practitioner outcomes and economic outcomes [43]. Patient health outcome measures of interest included, physical health and treatment outcomes (e.g. mortality, morbidity and surrogate physiological health measures) and psychosocial outcomes (e.g. quality of life) [43]. Practitioner/process outcome measures related to quality of care provided by community pharmacy. This included adherence to recommended practice or guidelines (e.g. extent to which health care providers gave appropriate advice, delivered clinical interventions and followed referral guidelines) [43]. Economic outcomes calculated resource use and measured costs and cost savings associated with guideline implementation (e.g. human resources, consumables and equipment) [43]. Secondary (surrogate) outcomes are those that may indirectly reflect important outcomes, including measures such as knowledge, attitudes and satisfaction of both patients and practitioners [43]. Also of interest was how outcomes were determined. Objective measures involve an impartial measurement and are usually considered more reliable than self-reported measures, which are subjective [44]. Examples of objective data include information from medical or health records, while subjective data may be obtained from a self-report questionnaire.

### Data abstraction, synthesis and quality assessment

A comprehensive data abstraction table was developed based on two standard checklists: The Cochrane data collection form (for intervention reviews of randomised controlled trials and non-randomised controlled trials) and the transparent reporting of evaluations with non-randomised designs (TREND) statement checklist (for non-randomised evaluations of behavioural and public health interventions) [45, 46]. The data abstraction table was piloted initially using two articles and revised by consensus (KW and HW). Using the revised table, data abstraction was completed independently by two authors (KW and HW). Discrepancies in data abstraction were resolved through discussion, and where consensus could not be achieved, by mediation with a third author (CS).

A narrative synthesis was undertaken because the methodological and clinical heterogeneity of the studies in this review determined that meta-analysis was not appropriate. A narrative synthesis satisfied the aim by considering different implementation strategies and determining their effectiveness in achieving outcomes reflecting the benefit of clinical guidelines in the community pharmacy setting.

Risk of bias was assessed for all studies included in the review. Studies that were either randomised controlled trials (RCTs), non-randomised controlled trials (NRCTs) or controlled before and after studies (CBAs) were evaluated for bias using the EPOC risk of bias tool [47]. Other studies in this review were cohort or quasi-experimental studies and were evaluated for quality using the Newcastle-Ottawa quality assessment scale for cohort studies [48]. These tools were chosen based on the Cochrane recommendation to use a domain-based evaluation for risk of bias assessments, rather than a tool with a summary score [49]. The Newcastle-Ottawa tool was one of only two tools recommended by Deeks in a review of 182 instruments for measuring risk of bias in cohort studies [50].

The Grading of Recommendations Assessment, Development and Evaluation working group (GRADE) approach [51] was used, in conjunction with the risk of bias tools, to evaluate quality of evidence for outcomes. This is because risk of bias can vary across outcomes, a consideration which is often ignored in systematic reviews [52]. To complete the GRADE assessment, worksheets were used to prepare a summary of findings table [53].This involved a three-step process of assessing the relative importance of all study outcomes, assessing the certainty of evidence across studies for an outcome and summarising the findings [53].

All quality evaluations including risk of bias and quality of evidence for outcomes were completed independently and then by consensus of two authors, KW and HW, with mediation by CS when required.

A critical appraisal of economic outcomes and economic modelling was not undertaken as it was beyond the scope of this review.

## Results

### Study selection

Searching the databases resulted in a total of 1937 articles. After adjusting for duplicates and non-English language articles, 1446 remained. Title and abstract screening excluded all but 36 articles. Examining the full-text records and reference lists of the remaining 36 articles resulted in 19 articles reporting on 22 studies, meeting the inclusion criteria. Five articles were discussed with the moderator to achieve consensus (Kappa score 0.86) Fig. 1.

### Study characteristics including risk of bias assessments

The 22 studies comprised 10 RCTs [54–63], 3 NRCTs [64–66], 1 CBA trial [67] and 8 quasi-experimental or observational studies [68–75] (Tables 1 and 2). The studies were conducted in Australia (*n* = 8) [54–56, 60, 64, 65, 68, 69], the USA (*n* = 4) [57–59, 73], the UK (*n* = 3) [62, 63, 75], the Netherlands (*n* = 2) [61, 72], Belgium (*n* = 1) [67], Canada, (*n* = 1) [71], Finland (*n* = 1) [74], Germany (*n* = 1) [70] and Switzerland (*n* = 1) [66]. The time frame for intervention-duration ranged from 1 day to 2 years. The included studies had sample sizes ranging from a group of 8 pharmacists to an intervention group of 1222 community pharmacies. Power calculations to determine an appropriate sample size were performed in three studies [62, 66, 70].

The 22 studies included in the review were assessed for risk of bias using separate tools depending on the study design. Consensus was achieved through discussion. Moderation was required for one domain (“comparability domain” in the Newcastle-Ottawa tool), which was the main area of disagreement (Tables 3 and 4).

Fourteen studies were assessed using the EPOC risk of bias tool for RCTs, NRCTs and CBA studies. Of these, 11 studies were evaluated as having a high risk of bias [55–59, 61, 63–67] and three studies assessed as having an unclear risk of bias [54, 60, 62]. Several domains contributed to risk of bias in multiple studies. These included, inadequately addressing incomplete data, lack of similarity in baseline measurements and protection from contamination in the study. It was not clear if these inadequacies were due to lack of reporting or flaws in research design. Two of the studies evaluated as having an overall unclear risk of bias used implementation strategies involving a computer prompt [54, 60].

The Newcastle-Ottawa tool for cohort studies was used to evaluate eight studies [68–75]. The study by Egen showed the least risk of bias with the domains around selection of study groups and outcomes deemed low risk [70]. However, like all of the cohort studies, in this review, the study design or analysis meant the comparability of cohorts could be a source of bias. There was little to suggest that studies had used measures to correct for confounding variables in any of the eight studies evaluated.

Use of the two risk of bias tools lead to the observation that many of the studies in this review were subject to selection bias and performance bias. Selection bias was inherent in the self-selection of study participants. Self-selection of participants occurred in 18 of the 22 studies, even if randomisation methods were subsequently used [54–58, 60–64, 66–69, 71–74]. Performance bias relates to the propensity for the studies to be threatened by the Hawthorne effect, which is the tendency to modify behaviour when being observed [76, 77]. Simulated patient methodology is one method that has been used in pharmacy-practice research to avoid the Hawthorne effect [78]. In this review, simulated patient methodology was used in ten studies [55, 56, 62–64, 66, 68–70, 75]. However, in most instances, the studies used the simulated patients overtly rather than covertly, which while ethically sound, does not eliminate the risk of bias.

### Implementation strategies and their effectiveness

The areas of clinical practice that guideline implementation were designed to impact were varied. They involved chronic disease states [57, 60, 65, 71, 75], guidelines for the supply of particular classes of medications [55, 56, 59, 61, 64, 66, 68–70, 72], community pharmacy’s role in preventative health [58, 67, 73] and guidelines for appropriate pharmacy practice [63, 74]. The variation did not allow for any conclusions to be made about the effectiveness of implementation based on guideline characteristics.

Implementation activities were targeted to community pharmacy, and in particular to pharmacists in 20 studies [54–62, 65–75] and pharmacy support staff in 11 studies [56, 58, 61–64, 66, 68, 69, 72, 75]. In two studies, implementation activities were directed exclusively to pharmacy support staff, while three studies involved implementation activities to other health professionals and/or patients as well as community pharmacy staff [58, 65, 70]. Both the studies in this review involving comparisons between professional and support staff demonstrated that better outcomes were achieved when the patient encountered a pharmacist [62, 75].

Eighteen studies involved multifaceted interventions [54–58, 60, 62–70, 73–75], and single intervention strategies were used in four studies [59, 63, 71, 72]. The multifaceted nature of the majority of implementation strategies did not allow for a clear understanding of the effectiveness of individual strategies. Overall, eight different implementation strategies were reported in studies in the review including, use of educational materials, educational meetings, educational outreach visits, mass media campaigns, audit and feedback, reminders (including CDSS), practice support and fee for service.

The most commonly used implementation strategies were educational interventions. All but two of the studies in the review had an educational component in their intervention including provision of educational materials, educational meetings or educational outreach visits [54–59, 61–70, 72–75]. Seven studies used educational strategies exclusively [59, 63, 65, 72–75]. Of these, two studies used behaviour change theory to inform the intervention [63, 73] resulting in variable outcomes. The remaining five studies achieved modest positive outcomes. Educational outreach visits (sometimes called academic detailing) were undertaken in three studies [62, 65, 70]. One of the studies by Watson compared educational outreach and education meetings, individually and in combination, against a control [62]. This study did not demonstrate effectiveness for either strategy.

In six studies, educational interventions were combined with audit and feedback [55, 56, 64, 66, 68, 69]. Benrimoj and colleagues authored all of these studies. Five of the studies (from two papers) were in collaboration with De Almeida Neto [55, 56, 64, 68, 69] as the primary author, and one study was in collaboration with Sigrist [66] as the primary author. The studies using audit and feedback were generally effective. All reported positive outcomes except one, which had variable outcomes [66].

Practice support, in the form of follow-up visits, phone calls or via provision of practice tools (e.g. checklists, patient handouts, documentation forms), was used in seven studies [55, 56, 58, 60–62]. Only one study used practice support as a single intervention [71]. The study was a pilot study that provided pharmacists with practice tools for diabetes management. The proposed method was to implement the tools in conjunction with an education programme; however, the education programme was not available for the pilot study. The use of the practice support tools alone resulted in a modest positive outcome.

Computer prompts, as reminders to support practice change, were the used in four studies [54, 57, 60, 67, 71]. The studies using this implementation strategy in the community pharmacy setting produced variable outcomes. The sub-studies by Curtain and Reeve [54, 60], which were both part of the larger Pharmacy Recording of Medication and Services (PROMISe) project [79], demonstrated very strong evidence of effect, whereas the study by Kradjan [57] demonstrated no effect. The strongly positive outcomes and comparatively robust methodology, seen in the Curtain [54] and Reeve [60] studies, indicate the best evidence for effectiveness of an implementation strategy in the community pharmacy setting.

The study by Egen used an intensive mass media campaign along with specific educational interventions directed to pharmacists and gynaecologists [70]. This multifaceted approach did not demonstrate evidence of effect in pharmacist- or patient-outcome measures.

A monetary incentive (fee for service) was only used in one study as an implementation strategy [57]. However, it was part of a multifaceted implementation, which also involved educational interventions and reminders. There was no evidence of effectiveness demonstrated.

### Basis for implementation strategies

Most implementation strategies were educational and designed to improve knowledge and/or communication skills. However, this choice of implementation strategy was, in almost all instances, not based on an identified deficit in these areas. Tailored implementation strategies, based on addressing identified barriers to successful guideline implementation, were only evident in two studies [61, 63]. The barriers identified included, organisational barriers, knowledge deficits, social factors such as patient indifference and suboptimal communication by non-professional pharmacy staff. Both the studies, which used tailored strategies, were ineffective in producing positive primary outcomes. Two other studies also addressed organisational factors (including workflow and time constraints), but without mentioning that the choice of strategy was tailored [58, 71]. Both proved to be moderately effective. Studies were based on specific behavioural theorems or frameworks in six instances [55, 56, 58, 63, 66, 73]. The behavioural theories, frameworks and strategies used included “Motivational Interviewing” techniques [55]; “Stages of Change Model” [55, 56, 66]; “Trans‐theoretical Model for Change” [73]; “Social Cognitive Theory” [58]; “Health Beliefs Model” [66]; “Theory of Planned Behaviour” [63]; “Calgary-Cambridge Model of Communication Skills” [63] and “Cognitive Behavioural Therapy” [63] techniques. Despite most of the studies in this review reporting positive outcomes, two out of the six studies based on behaviour change models did not [63, 66].

### Characteristics of outcome measures including quality of evidence assessments

Fifteen studies [54–56, 58–60, 64, 65, 67–69, 71–73, 75] reported positive outcomes as a result of clinical guidelines implementation. Five studies indicated no significant improvement in primary outcomes measures as a result of the implementation strategy [57, 61–63, 70] and two studies reported minimal or variable results [66, 74]. Almost all studies reported multiple outcomes making assessment of primary outcomes difficult. Objective measures were used to report outcomes in 18 studies [54–56, 59–69, 71, 73–75]. Self-reported outcomes, both patient and practitioner outcomes, were included in 15 studies [54, 55, 57, 58, 60–63, 65, 67, 70–74]. In many instances, these outcomes were assessed via novel un-validated tools, modified validated tools or the reporting was inadequate to ascertain validity and reliability of the tool. Such measures are of limited benefit in evaluating successful guideline implementation. Surrogate measures were used in nine studies and included assessments of attitudes, self-efficacy, and patient and practitioner satisfaction/acceptability of interventions [55, 57, 60, 61, 65, 67, 71, 73, 74]. Four studies did not use any objective measures and relied upon self-reported (subjective) measures [57, 67, 71, 74].

All studies in the review measured process outcomes related to changes in the practice of community pharmacy staff. Patient outcomes were only measured in three studies and all relied upon self-reported outcomes using patient surveys [54, 57, 70]. Only one of the patient outcomes measured was based on a health outcome, but it was a surrogate measure for perceived asthma control [57]. The lack of measurement of robust (objective) patient outcomes determines that few conclusions can be made about the evidence of effectiveness for implementation of guidelines to community pharmacy.

Most studies did not comment on sustainability of outcomes or mentioned it as a limitation of the research. Sustainable practice change was noted in two studies, but the sample sizes were small [71, 73]. Four studies indicated a decline in outcomes over the course of data collection [54, 60, 61, 71]. One study indicated that ongoing communication was required for sustainability [75]. Thus there is little evidence that the positive outcomes generated by implementation of clinical guidelines in most of the studies, are sustainable effects.

Economic outcomes were determined in three studies [54, 59, 62]. All the economic evaluations looked at different measurements. These included the cost of implementing guidelines [62], cost savings to the health system due to improved guideline adherence [54] and an attempt at a more thorough cost-benefit analysis [59]. No analysis was undertaken to assess the quality of the economic modelling undertaken.

Six primary outcome measures were agreed upon and assessed using the GRADE approach [51]. Five outcomes were assessed as having very low quality of evidence. One outcome was assessed as having low quality of evidence. The main reasons for this were the heterogeneity of the studies, variability of outcomes and the potential for bias in studies. None of the outcomes were considered to have moderate or high quality of evidence. Thus, even though the studies demonstrated positive outcomes from clinical guideline implementation, these outcomes are generally not a reliable indication of the likely effect. The likelihood that the effect will be substantially different is very high (Table 5).

## Discussion

This systematic review expands on the limited research into guideline implementation to allied health practitioners [13, 16] and the extensive research on guideline implementation to medical practitioners in hospitals and the primary healthcare setting [1, 2, 25, 26]. The findings of this review indicate that there is a growing body of evidence on clinical guideline implementation to community pharmacy, but conclusions that can be drawn from this evidence are limited. Many studies lack rigour in their methodology and are at risk of substantial bias. There is also a great deal of variability in the studies in this field making analysis challenging. The studies to date generally do not provide evidence of a grounded approach to the development of their implementation strategies. The strategies employed are mostly multifaceted, with an over-reliance on educational interventions. Reporting of multiple outcomes complicates assessment of the effectiveness of clinical guidelines implementation. The focus for outcomes is on process and surrogate outcomes, rather than patient outcomes, and the quality of evidence for outcomes is low. However, despite the limitations in the research to date, there are indications that clinical guideline implementation may be of benefit and that CDSS may be an effective implementation strategy in this setting.

In this review, variability was seen in the types of guidelines and areas of clinical practice, the intervention strategies utilised and the resultant outcomes, despite remaining focused in the community pharmacy setting. Other reviewers in implementation science have found similar variability. A recent systematic review, which only looked at the implementation of asthma protocols, noted inconsistent results in practitioner and patient outcomes within the one disease state [80].

The lack of rationale in intervention design and over-reliance on educational implementation strategies are also not unique to research in community pharmacy. There is a similar over-reliance on educational interventions in the literature [17, 18, 22]. This is surprising because educational interventions have been demonstrated to be minimally effective, particularly if they simply involve passive dissemination of information [2, 18, 22, 81]. This review demonstrated a similar minimal effectiveness of educational strategies, although it was hard to determine due to the multifaceted nature of most interventions. As the evidence in the literature for a multifaceted approach remains inconclusive, it would seem sensible, wherever possible, to use less complex interventions [14]. Potentially, less complicated interventions would be more cost effective, easier to sustain and better able to inform future practice [14].

A clear rationale involves using a tailored approach or theoretical framework to inform the implementation strategy.

Current consensus in the literature is that implementation strategies should consider the barriers and facilitators (determinants) of change [2, 16–18, 25, 82]. Although researchers agree on the use of a tailored approach, there are currently no recommended, reliable ways to identify and overcome barriers to successful implementation [22, 82]. Krause et al. demonstrated the complexity of tailoring implementation strategies in the area of chronic disease management [82]. The complexity of devising a tailored strategy is what may have resulted in the small number of studies assessing barriers to implementation in this review. It may also explain the lack of effectiveness of tailored implementation strategies observed, which seems to challenge the recommendations in the literature. Community pharmacy is a complex, retail environment with many staff and possibly many barriers to clinical guideline implementation. A greater understanding of the barriers unique to the community pharmacy setting may improve outcomes.

The current literature also promotes the use of theory to underpin study design, but with little knowledge of how to choose between the many behaviour change theories and how to successfully translate their constructs into an intervention [20, 21, 23, 83]. It is these challenges that may have resulted in very few studies in this review using a theory-based approach to implementation. Furthermore, the studies in this review that did use theory to guide implementation produced variable results. This is also consistent with the literature. While there are strong advocates for the use of theory in implementation science, interventions based on theory are not necessarily more effective [84]. However, there are potential advantages to the generalisability and replicability of theory-based implementation interventions [20], which would be useful to improving the evidence for guideline implementation in community pharmacy.

The implementation strategy to show the most promise in the community pharmacy setting was CDSS. Two studies using this strategy measured objective outcomes and demonstrated a strong effect, as well as being comparatively rigorous in their methodology. This observation holds logical appeal because CDSS has the potential to integrate with the existing workflows of pharmacists [85]. As pharmacists routinely use computers in the dispensing process, CDSS reminders possibly have fewer barriers to overcome in terms of integration into practice. Legrand et al. demonstrated the superiority of computer-integrated reminders compared to non-integrated information in their study on medicines and driving [67]. However, further research is required to truly understand the potential of computer prompts in community pharmacy: what types of prompts are effective, what variables influence effectiveness, the sustainability of such interventions and ultimately their effect on patient health outcomes. Supporting the evidence of benefit for CDSS in the community pharmacy setting is the fact that reminders have improved outcomes in other settings [2, 25, 86].

While the majority of studies in this review reported positive outcomes, there was variability in the types of outcome measures used and the magnitude of the effect, both between and within studies. These findings are consistent with the current literature for both allied health and the medical profession [2, 6, 13, 16, 20]. Multiple outcome measures in studies were common, and this added to the challenge of interpreting the results. Interpretation of these results also requires consideration of the quality of evidence and whether the research outcomes are valid measures of clinically significant changes in practice and patient care.

Detection of patient health outcomes can be difficult in the time frames seen for most studies in this review. Also, many of the studies implemented guidelines for the treatment of minor ailments using non-prescription medications. The expected clinical benefit in such instances would be small and challenging to measure. Worrall and colleagues support this notion with their review, which found little evidence to suggest that clinical practice guidelines produce significant changes in clinical outcomes in primary care [26].

Understandably, very few of the studies in this review looked at patient outcomes, and only one study measured a patient health outcome, which was a surrogate measure about perceptions of asthma control using a non-validated tool [57]. Consequently, no objective health outcomes were measured in this review. Given that improving patient health outcomes is the main objective in the development and implementation of clinical guidelines, the current research in community pharmacy is failing to provide substantive evidence of effect. Researchers have noted a similar lack of patient outcome data regarding clinical guideline implementation in other settings and professions [2, 16, 26, 87]. The main outcome measures seen in the literature [87] and in this review were process measures, at the level of the practitioner.

All the studies in this review assessed practitioner behaviour change, and most used objective measures to assess practice. While such measures may indicate behaviour change on the part of the practitioner, they do not necessarily indicate successful guideline implementation, translation of evidence into practice and may not correspond with patient outcomes.

One of the more interesting observations from this review was the importance of considering the role of support staff in the community pharmacy setting when implementing guidelines. Two studies in this review measured outcomes achieved when strategies were directed at professional pharmacy compared to support staff. Both studies demonstrated that better outcomes were achieved when the patient encountered a pharmacist. [62, 75]. This observation has also been substantiated in other pharmacy practice research [88, 89]. The implications of this surrogate outcome measure lie more in the consideration of study design in the community pharmacy setting. It is possible that lack of acknowledgement of the influence of pharmacy support staff, in designing an implementation intervention, could be responsible for some of the variability in outcomes observed.

Another major problem that limits the ability of researchers to make firm conclusions about guideline implementation is the poor methodological quality of studies and the poor reporting of interventions [2, 10, 13, 22, 26, 76, 90]. Unfortunately, this review is no different. Many studies used self-selection in the recruitment process. Self-selection results in samples that are unlikely to be representative of the wider population [91]. This selection bias leads to recruitment of participants who are motivated and more likely to adopt practice changes [91]. Performance bias was also inherent in many studies in this review. Unfortunately, by its very nature, practice-based research is vulnerable to participant-behaviour change due to the consent and awareness created by the research [13]. For controlled trials, selection and performance bias occur in both intervention and control groups. This can lead to small differences in effect between groups, which perhaps underestimates the value of the implementation strategy, but it may also overestimate the absolute effect of an intervention [91]. The concern is that findings do not necessarily translate to the “real world” [9, 87, 90].

## Strengths

The strength of this review is that it focuses on the single organisational setting of community pharmacy, which is unique. A criticism of implementation science has been that it can be hard to decipher the components of an intervention and the basis for using a strategy [92]. This review has managed to detail these characteristics for each study, providing a greater understanding of the gaps in research. A comprehensive assessment of the quality of all included studies and primary outcomes, using three validated tools, also allowed for insight into the strength of evidence from the studies.

## Limitations

A limitation of this narrative synthesis is that of the 22 studies synthesised, half were from Australia or the UK, and there were multiple studies by only three research groups (Benrimoj, The PROMISe researchers and Watson) [54–56, 60, 62–64, 66, 68, 69]. When the weighting of evidence is clustered in such a way, there is a reduced ability to make generalised conclusions. Conclusions were also limited by the determination that most of the studies were at a high risk of bias. However, the authors of the papers in this review were not contacted for further information. Such information may have helped to better discern methodological quality and provided stronger evidence. Another concern is that the majority of positive outcomes were the result of publication bias. There are also limitations in a narrative synthesis compared to meta-analysis.

## Practice implications

The findings of this review make an important contribution to the evidence base for the role of clinical guidelines in the community pharmacy setting. The review has the potential to guide pharmacy practice research and inform successful implementation, of clinical guidelines, in community pharmacy in the future. As highly accessible primary healthcare practitioners, the scope of practice of community pharmacists is increasingly important in influencing patient health outcomes. In recent years, there has been a focus by governments and non-government organisations on primary healthcare due to the recognised benefits to patients and society that a strong primary healthcare system generates [28, 93, 94]. Improving the effectiveness of pharmacists to provide evidence-based care through the use of clinical guidelines can strengthen primary healthcare.

## Conclusion

While this review points to the potential of clinical guidelines to influence practice in community pharmacy, at present, there is little to suggest that they positively affect patient outcomes. There is also little evidence on the best strategies for implementation. This lack of evidence is not surprising due to the complexity of implementation science, the heterogeneity of studies and the poor methodological quality of research in this setting. In the future, study design should focus on using a more systematic approach. More attention should be given to the rationale of an implementation intervention and the choice of outcome measures. The community pharmacy setting is unique in the influence that pharmacy support staff can have on outcomes achieved by guideline implementation. As a result, careful consideration should be given to their role in any study design. Improved methodological rigour in future research will strengthen the evidence of the benefits of clinical guidelines and the best strategies to implement them in community pharmacy.

## Additional file

Additional file 1:
**Search terms.**

## Abbreviations

ADDOs: Accredited Drug Dispensing Outlets
CBA: controlled before-and-after Trial
CDSS: computerised clinical decision support system
EPOC: Cochrane Effective Practice and Organisation Care Review Group
GRADE: Grading of Recommendations Assessment, Development and Evaluation
MCAs: medicine counter assistant
NRCT: non-randomised controlled trial
PICO: participants, interventions, comparators, outcome framework
PRISMA: preferred reporting for systematic reviews and meta-analyses
RCT: randomised controlled trial
TREND: transparent reporting of evaluations with non-randomised designs
