# Supplementary material for: Effectiveness of implementation strategies for clinical guidelines to community pharmacy: a systematic review
Source: Implement Sci. 2015 Oct 29;10:151. doi: 10.1186/s13012-015-0337-7 (PMC4627629; doi:10.1186/s13012-015-0337-7)
Supplement: Additional file 1: — Search terms. [file 13012_2015_337_MOESM1_ESM.doc]

**Appendix 1: Search Terms**

**^#^** The search term within each column were combined with “OR” and between column were combined with “AND”

| **MEDLINE:** | | |
| --- | --- | --- |
| Pharmacy [MeSH] | Health Plan Implementation [MeSH] | Guideline [MeSH] |
| Pharmacists [MeSH] | Information Dissemination [MeSH] | Guideline Adherence [MeSH] |
| Community Pharmacy Services [MeSH] | Education, Pharmacy [MeSH] | Practice Guideline [MeSH] |
| Community pharmac*.mp | Implement*.mp | Evidenced Based Medicine [MeSH] |
| Drug store*.mp |  | Algorithms [MeSH] |
|  |  | Clinical Protocols [MeSH] |
|  |  | Quality of Health Care [MeSH] |
|  |  | Guide*.mp |
|  |  | Guideline implementation.mp |
|  |  | “Standards of Care” [MeSH] |
| **Cochrane Library:** | | |
| Pharmacies [MeSH] – term only | Health Plan Implementation [MeSH] – explode all trees | Guidelines as Topic [MeSH] – explode all trees |
| Pharmacists [MeSH] - term only | Information Dissemination [MeSH] – explode all trees | Guideline Adherence [MeSH] – explode all trees |
| Community Pharmacy Services [MeSH] - term only | Education, Pharmacy [MeSH] - term only | Practice Guideline as Topic [MeSH] – explode all trees |
| Community pharmac*.kw | Implement* | Evidenced Based Practice [MeSH] – explode all trees |
|  |  | Algorithms [MeSH] – explode all trees |
|  |  | Clinical Protocols [MeSH] – explode all trees |
|  |  | Quality of Health Care [MeSH] – explode all trees |
|  |  | Health Planning Guidelines [MeSH] – explode all trees |
|  |  | Guide*.kw |
|  |  | Guideline implementation*.kw |
| **EMBASE:** | | |
| Pharmacy [MeSH] | Information Dissemination [MeSH] | Practice Guideline [MeSH] |
| Pharmacist [MeSH] | Allied Health Education [MeSH] | Evidenced Based Medicine [MeSH] |
| Community pharmac*.mp | Clinical Education [MeSH] | Algorithms [MeSH] |
| Drug store*.mp | Implement*.ab | Clinical Protocols [MeSH] |
|  |  | Health Care Quality [MeSH] |
|  |  | Guideline implementation*.mp |
|  |  |  |
| **CINAHL Plus: 23 References** | | |
| Pharmacy and Pharmacology [MH] | Selective Dissemination of Information [MH] | Practice Guidelines [MH] |
| Pharmacist [MH] | Education, Continuing [MH] | Algorithms [MH] |
| Community pharmac*.TX | Systems Implementation [MH] | Protocols [MH] |
| Drug store*.TX | Program Implementation [MH] | Quality of Health Care [MH] |
|  |  | Quality Improvement [MH] |
|  | Implement* TX | Guideline implementation*TX |
| **Web of Science:** | | |
| “Community Pharmac*”Topic | Implement* Topic | Guideline*Topic |
| “Retail Pharmac*” Topic | Disseminat*Topic | “Evidence based*” |
| Drug store*Topic |  | Algorithm*Topic |
|  |  | Protocol*Topic |
|  |  | “Professional Standard*”Topic |
| **Informit:** | | |
| Pharmac* | Implement* | Guide* |
| Community pharmac* | Disseminat* | Practice guide* |
|  | Educat* | Evidence based* |
|  |  | Algorithm* |
|  |  | Clinical protocol* |
